# Development and evaluation of couple-based coping strategies with infertility: a protocol for a mixed-methods study

**DOI:** 10.1186/s13063-022-06795-8

**Published:** 2022-10-04

**Authors:** Marzie Reisi, Ashraf Kazemi, Mohammad Reza Abedi

**Affiliations:** 1 Student Research Committee, School of Nursing and Midwifery, Isfahan University of Medical Sciences, Isfahan, Iran; 2 Reproductive Health Department, School of Nursing and Midwifery, Isfahan University of Medical Sciences, Hezarjerib AV, Isfahan, Iran; 3 Counseling Department, School of Psychology and Education Sciences, University of Isfahan, Isfahan, Iran

**Keywords:** Infertility, Coping strategies, Couple approach

## Abstract

**Background:**

Different coping strategies have been associated with various effects on infertile couples’ mental health. Considering the close interaction between couples, the present study aims to develop coping strategies for infertile couples using a couple-based approach.

**Methods:**

The present mixed-methods study will be conducted in three phases. In order to develop coping strategies, a qualitative study will be conducted in the first phase, during which semi-structured interviews will be implemented with infertile couples in order to collect data related to their lived experiences in using coping strategies. These data will be inductively analyzed using qualitative content analysis and interpretative phenomenological analysis. In the second phase, using the Delphi method, an initial draft of coping strategies will be designed using a couple-based approach based on the data obtained from the qualitative study and the related literature review. In the third phase, the designed couple-based strategies will be provided to two groups of infertile couples as a randomized field superiority trial study. The couples’ levels of stress, anxiety, depression, and adjustment will be evaluated using validated questionnaires before and 3 months after the intervention.

**Discussion:**

Couple-based coping strategies encourage couples to become aware of each other’s feelings while interacting and choose a strategy based on such feelings.

**Trial registration:**

Iranian Registry of Clinical Trials IRCT20191014045102N1. Registered on 19 October 2021. Protocol version: Current protocol: version 1 (22 May 2022)

## Background

Infertility affects approximately 10 to 15% of couples of reproductive age worldwide [1, 2]. This experience is associated with a wide range of psychological problems for couples such as depression [3, 4], stress, anxiety [5, 6], low self-esteem [7, 8], and low psychological adjustment [9]. On the other hand, social pressures due to the infertility taboo can create a psychological imbalance between couples and, in some cases, weaken their relationships [10]. Domar et al. believe that infertility is a source of chronic stress and should be seriously taken into account as much as major physical health problems, such as cancer and coronary heart diseases [11]. Infertility is considered a crisis by most couples [12], and adaptive strategies under infertility stress are associated with adjustment and reducing the psychological burden of infertility [13].

Most couples choose coping strategies to deal with infertility and mitigate the resulting mental pressure. Coping strategies are a set of cognitive and behavioral efforts made to interpret, analyze, and modify a stressful situation [14]. Lazarus defines coping as a response to mental pressure such as infertility. Such a reaction is a personal attempt to overcome harmful, threatening, or challenging circumstances [15].

Coping strategies are classified into four groups: active coping, active avoidance, passive avoidance, and meaning-based coping [16]. The efficiency of any coping strategy depends on the controllable or uncontrollable conditions and social and cultural context of the society. For instance, some coping strategies to deal with maladjustment and psychological problems could affect inversely in various cultural environments [13]. Furthermore, couples’ close and comprehensive interactions can cause each spouse to be affected by their partner’s choice of coping strategies and psychological damage resulting from infertility. A coping strategy employed by each spouse could also influence their partner’s mental health [17].

However, the existing coping strategies are mainly focused on the individual. Coping strategies that can reduce an individual’s infertility stress may negatively impact the spouse’s mental health. Therefore, considering the interactive effect of couples’ coping strategies, it is essential to identify coping strategies whose interactive effect outcome is accompanied by a reduction in the psychological burden of infertility in both men and women, particularly in situations where men and women do not equally bear the psychological and social burden of infertility. In traditional societies where infertility is mainly attributed to women, they are more vulnerable to the social stigma of infertility than men [18].

Therefore, the present study intends to develop coping strategies for infertile couples using a couple-based approach and evaluate the effect of the designed coping strategies on infertile couples’ mental health. This trial will be a randomized field trial, superiority framework, with two parallel groups. The study objectives are as follows:

- Comparison of the depression, anxiety, and stress levels in the intervention and control before and after the intervention
- Comparison of the infertility adjustment in the intervention and control before and after the intervention

## Methods

This mixed-methods sequential exploratory study will be conducted in three phases with the permission of the Ethics Committee of Isfahan University of Medical Sciences in Isfahan, Iran. This study will be conducted in three phases. The first stage will consist of data collection and preparation of lists of couple-based coping strategies and includes qualitative study and advanced literature review. The second stage will include the Delphi method and assess the feasibility of the designed coping strategies in a randomized field trial study.

### First phase: designing the couple-based coping strategies

The qualitative phase of the study will be conducted by inductive content and interpretative phenomenological analyses in order to explain the infertile couples’ lived experiences of using coping strategies. At this stage, to collect data, the researcher will use in-depth and semi-structured interviews with infertile couples individually and in pairs. Guiding questions will be designed for in-depth interviews and used in semi-structured interviews. To collect data, voice recording, note-taking, and field notes will be used. This stage of the study will be conducted in comprehensive health centers and infertility centers in Isfahan.

#### Participants and setting

In the first phase of the study, participants will include Iranian infertile couples who underwent infertility treatment at Isfahan Fertility and Infertility Center. The couples will be selected using the purposeful sampling method with maximum diversity in terms of age, education levels, economic status, job, duration of infertility, type of infertility, cause of infertility, and level of adjustment with infertility. The inclusion criteria will include no history of mental disorders and at least 2 years of infertility.

#### Data collection

The participants will be selected using the purposeful sampling method. In order to invite the participants, the study objectives will be explained, and the interview time and setting will be determined. Participants’ details will be recorded before the interview. Moreover, the fertility adjustment scale will be completed by participants to ensure sampling with maximum diversity in terms of the adjustment with infertility.

In a face-to-face meeting, data will be collected through in-depth interviews, voice recordings, and field notes of the observations. Couples who are willing will participate in the interview as a pair. Considering the psychological conditions of the participants and their readiness to be interviewed, the interview may be postponed or stopped and resumed another day.

The interview will be unstructured to allow the discovery of new ideas and will be continued using semi-structured interviews. An interview will be developed containing a list of questions to be asked during the interview with the participants. To further deepen the interview and gain richer data, some exploratory questions such as “Could you explain this through more examples?” will be used. Data collection will be continued until data saturation.

#### Data management and analysis

These data will be inductively analyzed using qualitative content analysis and interpretative phenomenological analysis introduced by Smith et al. [19]. The process of the interpretative phenomenological analysis will include reading, re-reading, noting, finding patterns across the data, and interpreting the data. Each interview and the related observations will be considered as a research unit. The recorded interviews will be listened to and transcribed. Themes from all transcriptions will be juxtaposed and clustered together across the cases. The subordinate themes will be collated and extracted.

To evaluate the validity and reliability of the data, credibility, dependability, conformability, and transferability standards will be used as the scientific accuracy criteria. In order for credibility, the member checking technique will be used to verify the trustworthiness of the data and codes. In order to ensure dependability, initial codes and examples of the categories, themes, and items extracted from the texts of the interviews for each category will be provided to an external observer. To ensure confirmability, the text of a number of the interviews and extracted codes and categories will be provided to research colleagues and three reproductive health and psychology experts, and they will be asked to evaluate the accuracy of the data coding process. In order to ensure the transferability of the findings, the participants’ statements will be presented as objectively as possible.

### Second phase: Delphi method

At this stage, an initial draft of couple-based strategies will be confirmed and validated. In the first round, to prepare an initial draft of couple-based coping strategies to deal with infertility as well as a questionnaire of open-ended items, firstly, the qualitative data will be analyzed; secondly, couple-based coping strategies to deal with infertility will be identified and extracted; thirdly, advanced reviews of related literature and document will be implemented. In order to improve, the initial draft will be sent (either by email or post) to 10 specialists, including psychologists and reproductive health specialists, to inquire about their opinion on the developed coping strategies.

In the second round, strategies modified in the first round will be ranked in a checklist using a Likert scale. In this round, moreover, coping strategies approved or disapproved by experts will be separated. In the checklist, there will also be a section for the participant experts to express their new ideas, correction, interpretation, and deletion; explain the strengths and weaknesses of the strategies; and elaborate on the reason for prioritization of coping strategies. The checklist will be sent to the experts to complete. The data will be reviewed and analyzed after receiving the completed checklists.

In the third round, the final checklist of couple-based coping strategies to deal with infertility will be prepared based on experts’ opinions in the second round and sent to the study experts to ask for their opinion. Furthermore, in this phase, the participants will be asked to review their answers, revise them if necessary, and present their reasons for any disagreement. The findings of both the qualitative study and the Delphi panel will be triangulated to design the field trial.

### Third phase: a randomized field trial study

#### Participants and setting

This parallel-group, double-blind, controlled, randomized field trial, superiority framework with an allocation ratio of 1:1 will be conducted on infertile couples who underwent infertility treatment in Isfahan Fertility and Infertility Center, Isfahan, Iran. Couples, at least one of whom suffers from mild to moderate depression, will be invited to participate in the quasi-experimental study. The inclusion criteria will include no history of severe mental disorders and no stressful event during the last 3 months. The exclusion criteria will be a stressful event or the onset of assisted reproductive treatment during the intervention and ovarian hyper-stimulation syndrome due to ovarian induction.

#### Pilot study/feasibility

A pilot study will be carried out to estimate the number of couples required for initial screening, attrition rate, and the rate of reduction in postoperative depression. This pilot study will be performed on 50 infertile women. After obtaining informed consent, the couple’s depression level will be measured using a standard questionnaire.

The sample size required for initial screening in the field is estimated based on the prevalence of mild to moderate depression. Furthermore, to assess feasibility, after receiving informed consent, the intervention will be performed on couples who are eligible for research, and their level of depression will be measured for the second time. The depression levels at the first and second assessments will be compared by the research supervisor. If the level of depression would be increased in the second assessment, the trial will be stopped.

If any problems are identified in the implementation of the intervention, the necessary changes will be applied by the research team after reaching a consensus. Couples participating in the pilot study will not participate in the clinical study, and the results of the measurements will not be included in the analysis.

#### Sample size

The sample size will be calculated for a 1:1 allocation ratio, considering presumed depression score reduction after the intervention, estimated sample loss, and 95% confidence level.

#### Recruitment

Couple’s eligibility assessment and screening will be performed by the Ph.D. candidate in reproductive health. During the first interview, X infertile couples will be invited, and the levels of depression, anxiety, stress, and infertility adjustment will be evaluated using validated questionnaires. After determining the eligible couples, X couples will be randomly selected and invited by the researcher to participate in the study. The study objectives and the way of participating in the study will be explained to couples who accept the invitation.

The informed consent will be obtained from eligible couples by a legal advisor in the infertility center. The representative of the Ethics Committee of the Isfahan University of Medical Sciences will supervise all stages of the research and will decide to end the trial.

#### Sampling and data collection

After determining the eligible couples, X couples will be randomly selected by the researcher. To randomly assign the couples to the intervention and control groups, X closed envelopes containing blue and green cards will be prepared. The couples will be referred to the midwife consultant and receive one of the envelopes and complete demographic characteristics.

The midwife and participants will be blinded to the study group. After random allocation, one researcher, who will not be involved in sampling and random allocation, will assign each group’s card color. Couples’ depression, anxiety, stress, and infertility adjustment levels will be evaluated using valid questionnaires 2 and 3 months after starting the intervention and compared between the groups (Table 1).

**Table 1:** Schedule for data collection during the trial

Time point	Study period				
	Enrollment	Allocation	Post allocation		Closeout
	Pre-baseline	Baseline	Time 1	Time 2	
Enrollment					
Eligibility screen	X				
Informed consent	X				
Allocation		X			
Participant characteristics					
Demographics		X			
Infertility profile		X			
Interventions					
Couple-based coping strategies		X			
Solution-oriented counseling		X			
Outcomes assessment					
Depression			X	X	X
Anxiety			X	X	X
Stress			X	X	X
Infertility adjustment			X	X	X

#### Intervention

The intervention will begin before starting medical treatment. The counselors will be MSc holders in psychological counseling. They will be trained in the developed couple-based coping strategies using face-to-face education and a role modeling approach.

For the intervention group, a counseling psychologist will be educated on the developed couple-based coping strategies based on the color chosen for one group (intervention group) and solution-oriented counseling for the other group (control group).

The number, duration, and intervals of the sessions for intervention and other educational intervention (control) groups will be set identically. It is estimated that the counseling program will be scheduled 3 to 5 times once or twice each week. The researchers will bear the intervention and transportation costs for both intervention and control groups. Participants should not participate in any other research during the intervention. The research is expected to proceed without any harm; however, in case of harm, the main researcher will be responsible for compensating for the reparable harm.

#### Outcomes

The outcome will be the mean score of depression, anxiety, stress, and infertility adjustment and the magnitude of their change on enrollment and 3 months after starting the intervention in couples in the intervention group compared with those in another educational intervention (as the control group).

#### Data collection tools

Data will be collected using a self-report 21-item Depression Anxiety Stress Scale (DASS-21) and a revised version of the Fertility Adjustment Scale (R-FAS) [20]. Each of the DASS-21 subscales contains seven items on a 4-point Likert scale (0–3) from 0 (not at all true for me), 1 (to some extent true for me), 2 (considerably true for me), to 3 (absolutely true for me). A higher score indicates greater depression, anxiety, and stress.

Adjustment to infertility will be assessed using the self-report R-FAS, including 12 items on a 6-point Likert scale (1–6) from 1 (strongly disagree) to 6 (strongly agree). The final score will be obtained by summing the scores of questions; a higher score indicates a lower adjustment toward infertility.

To promote participant retention and complete follow-up, prior to each counseling session, the researcher will encourage couples who discontinue or deviate from intervention protocol to participate in counseling sessions through a telephone call. Missed data will be completed by the researcher through a telephone call and questions. Data monitoring in all phases will be done by the principal investigator.

#### Confidentiality

Researchers directly will be accountable for relevant information about the trial to the potential participants. When entering data in the datasheet, participants’ details will be removed, and the code will be assigned.

#### Data management and analysis

Data will be analyzed using the SPSS 19 software and statistical tests, including the independent *t*-test, pair *t*-test, chi-square, and repeated measures analysis of variance (RM ANOVA). If the measurements at the enrollment phase appear differently in the groups, analysis of covariance (ANCOVA), controlling for baseline data and independent variables (group as a fixed parameter and enrollment mean scores as a covariate), will be used. Also, the baseline value of outcome variables and potential confounding variables which are different between the two groups will be adjusted to avoid potential risk of bias and detect independent results. The data normality will be evaluated through the Kolmogorov-Smirnov test. If the data are not normally distributed, nonparametric tests such as Wilcoxon will be used. In the case of missing data, we will utilize the last value carried forward method to impute missing values.

### Presentation of the results

All results of the trial without any restrictions will be published.

## Discussion

Given that coping strategies have dissimilar effects on infertile couples’ mental health, appropriate coping strategies need to be taught to promote their mental health. A study has indicated that strategies used by infertile couples are influenced by cultural and social values and norms of the society where they live [13]. It has also been emphasized that culture affects the way a particular gender would interpret infertility, the way they would respond to it, and even the strategies they would apply to deal with this issue [21].

Therefore, it is necessary to develop supportive programs in treating couples’ infertility according to their lived experiences facing infertility and their social and cultural context. In addition, the infertility crisis is an event that affects couples’ married life and interactions, management of which requires active and responsible participation of couples. As a result, the interaction between coping strategies of each couple does not increase the psychological burden of infertility on the other.

In an effort to provide psychological support to infertile couples, Zurlo has developed a model of mental health predictor variables in infertile couples undergoing treatment that demonstrates the importance of adaptive coping strategies for infertile couples’ mental health [22]. The effect of some counseling methods on coping strategies of couples undergoing assisted reproductive technology has also been evaluated. Rasoulzadeh Bidgoli reported that the use of the Collaborative Infertility Counseling Model was associated with an increase in adaptive coping strategies such as seeking social support and problem-focused coping strategies in women undergoing infertility treatment [23].

In a study by Czamanski et al., positive reappraisal coping intervention and problem-solving skills training in women awaiting assisted reproductive technology resulted in increased use of problem-focused strategy and decreased use of emotion-focused strategy [24].

One study evaluated the effect of Positive Adjustment Coping Intervention using a smartphone to provide psychological support to women undergoing infertility treatment and found that the program was associated with a reduction in women’s psychological burden during treatment [25]. Moreover, the positive effect of cognitive-behavioral interventions on stress reduction in women undergoing assisted reproductive technology has been reported [24]. These findings suggest that adaptive coping strategies can reduce the psychological burden of infertility. Therefore, it is necessary for the couple-based strategies developed in this study to focus on the interaction between couples in order to reduce the psychological burden resulting from infertility and treatment in couples undergoing the treatment.

In the present study, coping strategies used by infertile couples to interact with each other and improve their adjustment to infertility will be extracted by analyzing their lived experiences and evaluating their emotions and feelings toward infertility. Such strategies, extracted and designed based on society’s cultural and social contexts, will better meet the couples’ emotional needs compared to other strategies. When a strategy is used by a partner regardless of the other partner’s emotional and strategic needs, it may harm another partner’s mental health. Therefore, designing couple-based coping strategies based on the data of a qualitative study and the cultural conditions of society is expected to minimize the possible friction between strategies used by couples and improve their relationships.

## Data Availability

The datasets will be available from the corresponding author upon reasonable request.

## Abbreviations

DASS-21: 21-item Depression Anxiety Stress Scale
R-FAS: Revised Version of the Fertility Adjustment Scale
